# Silencing of quinolinic acid phosphoribosyl transferase (*QPT*) gene for enhanced production of scopolamine in hairy root culture of *Duboisia leichhardtii*

**DOI:** 10.1038/s41598-018-32396-0

**Published:** 2018-09-17

**Authors:** Pooja Singh, Ratnum Prasad, Rashi Tewari, Meraj Jaidi, Susheel Kumar, P. K. Rout, Laiq ur Rahman

**Affiliations:** 1 Plant Biotechnology Division, Central Institute of Medicinal and Aromatic Plants (CSIR-CIMAP), P.O. CIMAP, Picnic Spot Road, Lucknow, U.P. 226015 India; 2 Academy of Scientific and Innovative Research (AcSIR), CSIR-Central Institute of Medicinal and Aromatic Plants, Lucknow, 226015 India; 3 Chemical Science Division, Central Institute of Medicinal and Aromatic Plants, P.O. CIMAP, Picnic Spot Road, Lucknow, U.P. 226015 India; 4 Plant Molecular Virology Laboratory, CPMB Division, National Botanical Research Institute, Lucknow, U.P. 226001 India

## Abstract

Scopolamine is a pharmaceutically important tropane alkaloid which is used therapeutically in the form of an anesthetic and antispasmodic drug. The present study demonstrates enhanced scopolamine production from transgenic hairy root clones of *Duboisia leichhardtii* wherein the expression of *quinolinate phosphoribosyl transferase* (*QPT*) gene was silenced using the *QPT*-*RNAi* construct under the control of CaMV 35 S promoter. The *RNAi* hairy roots clones *viz*. P4, P7, P8, and P12 showed the enhanced synthesis of scopolamine with significant inhibition of nicotine biosynthesis. Optimization of culture duration in combination with methyl jasmonate elicitor in different concentrations (50 µM-200 µM) was carried out. Maximum synthesis of scopolamine had obtained from HR clones P7 (8.84 ± 0.117 mg/gm) on the 30^th^ day of cultivation. Conspicuously, elicitation with wound-associated hormone methyl jasmonate enhanced the yield of scopolamine 2.2 fold (19.344 ± 0.275 mg/gm) compared to the culture lacking the elicitor. The transgenic hairy roots cultures established with RNAi mediated silencing of *quinolinate phosphoribosyl transferase* gene provides an alternative approach to increase the yield of scopolamine in fulfilling the demand of this secondary metabolite.

## Introduction

*Duboisia leichhardtii* (family-Solanaceae) indigenous to Australia and New Caledonia contains not only pyridine alkaloids but also tropane alkaloids. The main alkaloids of *D*. *leichhardtii* are scopolamine and hyoscyamine, both of which therapeutically used in the form of anesthetic and antispasmodic drugs^1^. These two tropane alkaloids have reputed medical application as anticholinergic agents, acting on the parasympathetic nervous system by blocking the activity of neurotransmitter acetylcholine^2^. The compounds have high pharmaceutical value; however, due to higher physiological activities and fewer side effects, scopolamine is preferable over hyoscyamine^3^. Besides being produced in *Duboisia species*, scopolamine naturally produced in the young roots of many Solanaceous plants like *Hyoscyamus*, *Atropa*, and *Scopolia*. It later mobilized into the leaf tissue. However, the level of scopolamine in these species is often very low^4–6^. Due to its limited availability and lack of synthetic source, the worldwide market demand for scopolamine has increased^7^. To meet the demand, *in vitro* hairy root technology has been used in many Solanaceous plants through *Agrobacterium*-mediated genetic transformation to increase the production of tropane alkaloids^8,9^. Scopolamine content increased through the repeated selection of *Duboisia myoporoides* wild-type root lines, and in *Atropa belladonna*, *Duboisia leichhardtii* and *D*. *myoporoides* through the biotransformation^2,10^.

Several investigators have used metabolic engineering of tropane alkaloids to improve the conversion rate of hyoscyamine to scopolamine through overexpression of the downstream gene (*H6H)* in *Duboisia* hybrid, *Duboisia leichhardtii*, *Nicotiana tabacum*, *Atropa belladonna*, and *Hyoscyamus niger* in the last decade. *H6H* codes for the last enzyme, involved in the conversion of hyoscyamine to scopolamine^3,6,7,11,12^. Intriguingly however besides producing hyoscyamine and scopolamine, the roots of *Duboisia* species also synthesize nicotine and related pyridine alkaloids normally found in tobacco plant^8^. The biosynthetic pathways of tropane and pyrimidine alkaloids overlap in intermediate steps. Methylpyrrolinium cation is the common precursor for the nicotine and tropane alkaloids, which is catalyzed by different enzyme^12–14^. It could be possible to divert the methylpyrrolinium cation towards scopolamine production by down-regulating the *Quinolinic acid phosphoribosyl transferase* (*QPT*) gene through metabolic engineering. The QPT enzyme catalyzes the nicotinate mononucleotide biosynthesis, which subsequently combines with methylpyrrolinium cation to form nicotine. Antisense-mediated suppression of *QPT* gene causes the down-regulation of the QPT enzyme resulting in reduced nicotine production^15,16^. Diverting the carbon flux towards the synthesis of the desired product by suppression of competing pathways is a practical option.

In the background of this information, in the present study, we have used the RNAi approach to silence *QPT* gene in the nicotine biosynthesis pathway of *Duboisia leichhardtii* hairy root culture, so that methylpyrrolinium cation, for the nicotine and tropane alkaloid pathway is diverted towards scopolamine synthesis. We report an increased amount of scopolamine in such cultures, making this approach a viable one for producing this pharmaceutically important alkaloid.

## Results and Discussion

### Vector construction and hairy root induction

A total of 15 root lines had induced by infection of *Agrobacterium rhizogenes* containing *QPT-RNAi* construct at a frequency of 70%. Some root lines did not grow well and turned brown after some time. The sturdy and fast growing root lines were selected further for analysis. The slow growth of *Duboisia* root had also reported previously^11^.

**Figure 1:**
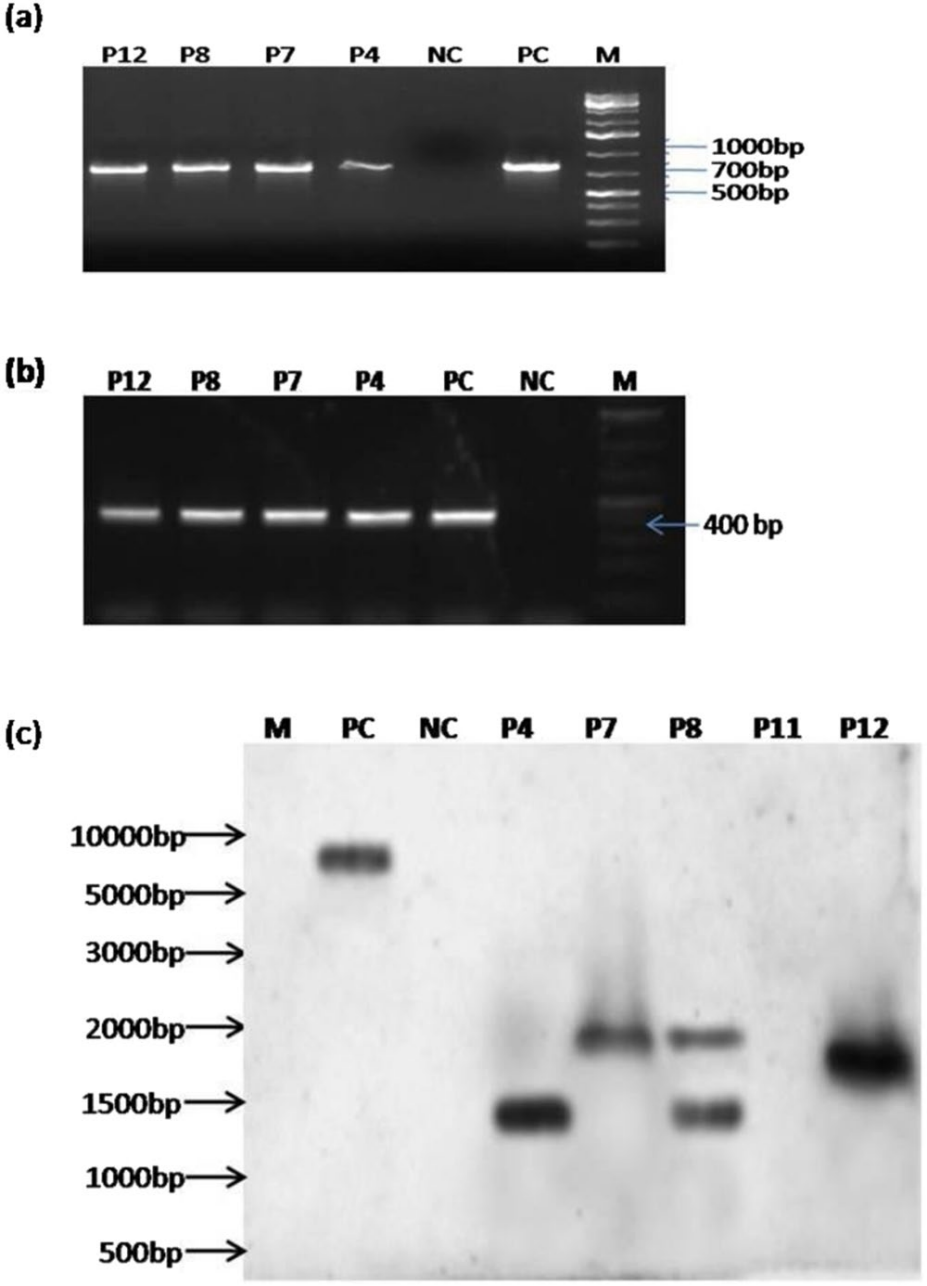
(**a**) PCR amplification of the *nptII* gene produced 786 bp of the amplicon. M- Marker (1 kb plus ladder), PC-Positive Control, NC-Negative Control. P4, P7, P8 & P12 are the putative transformants. (Full-length gel image presented in Supplementary Fig. S1). (**b**) PCR amplification of *rol* B gene produced 400 bp of the amplicon. M-marker (1 kb plus ladder), PC -Positive Control, NC -Negative Control, P4, P7, P8 & P12 are the putative transformants. (Full-length gel image presented in Supplementary Fig. S2). (**c**) Evaluation of putative transgenic hairy root lines by Southern hybridization. M-marker (1 Kb ladder), PC*-*Positive Control, NC- Negative Control, P4, P7, P8, P11, and P12 are putative transgenic hairy root lines. (Full-length gel image of the southern blot with the visible molecular marker have presented in Supplementary Fig. S3).

The presence of T-DNA, as well as insertion of the *QPT* gene with selection marker (*nptII*), was confirmed by PCR amplification of partial sequences of *nptII* and *rolB* gene respectively (Fig. 1a,b). Amplification of *rolB* indicates the transformed nature of the roots while the *nptII* gene specified the successful insertion of the *QPT* gene in four hairy root lines (P4, P7, P8, and P12). Southern blot analysis confirmed the insertion of one or multiple copies of the *nptII* gene (Fig. 1c). P4, P7 and P12 lines showed single copy insertion of the transgene in their genome while two copies inserted in the P8 hairy root. In P11 root lines insertion of the *nptII* gene was not observed.

### Growth kinetics, alkaloid production and expression level in *QPT-RNAi* hairy root cultures

**Figure 2:**
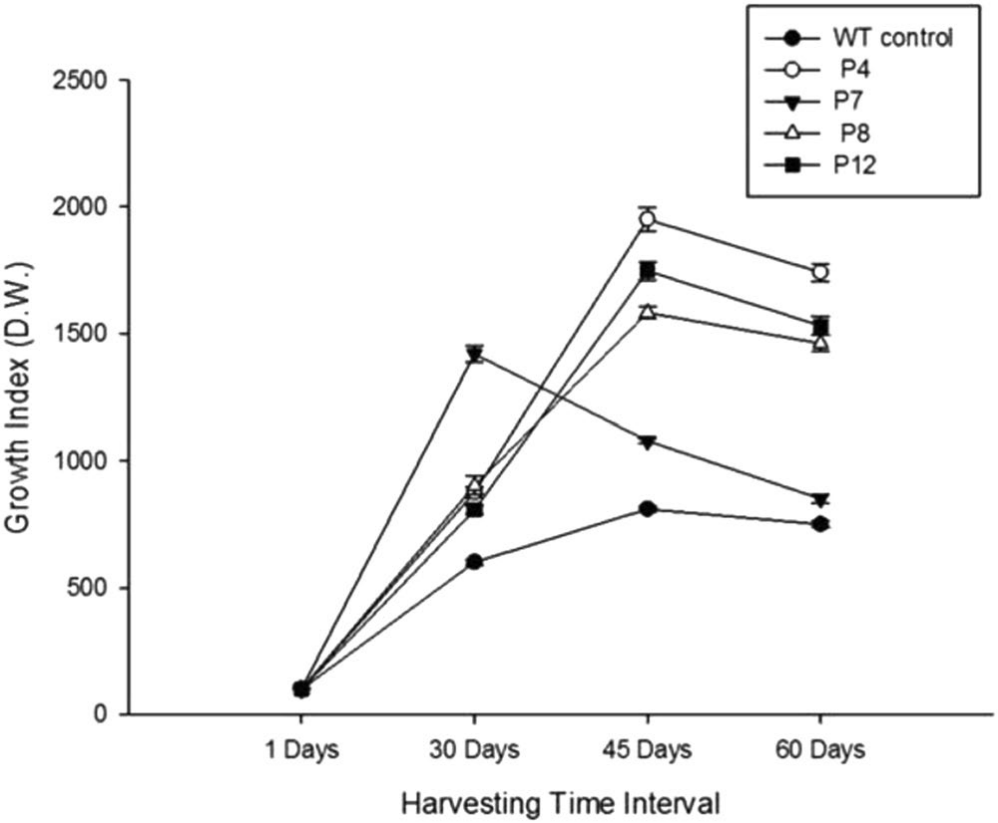
Growth kinetics of *D*. *leichharditii* hairy root lines (P4, P7, P8, and P12) at different harvesting time. Values are the means of three replicates ± S.D.

The root lines were subcultured in liquid MS medium every one month for two years to document the required stability of the transgenic lines^17^. Growth kinetics studies revealed that three root lines (P4, P8, and P12) and the control line attain their maximum growth at 45 days of culture in liquid MS media. However, P4 root line indicated comparatively better biomass yield than the P8, P12, and control (Fig. 2). The P4 line revealed ~19.5 fold increment over the initial inoculums, which was 2.4 fold higher than the control, resulting to its highest growth (GI = 1950.33 ± 45.45) at 45 days, followed by decline in growth potential (GI = 1741 ± 34.8) on advancement of the growth period up to 60 days (Fig. 2). In contrast, the P7 root line attained its maximum growth at 30 days (GI = 1419.33 ± 32), which revealed ~14 fold higher biomass than the initial inoculums (Fig. 2). Such a variation among hairy root clones was reported earlier and thought to be due to the different expression of T-DNA genes integrated at different locations in the genome of the transformed hairy roots^18^. Such ‘position effect’ mediated differences are common in transgenic plants^19^.

**Figure 3:**
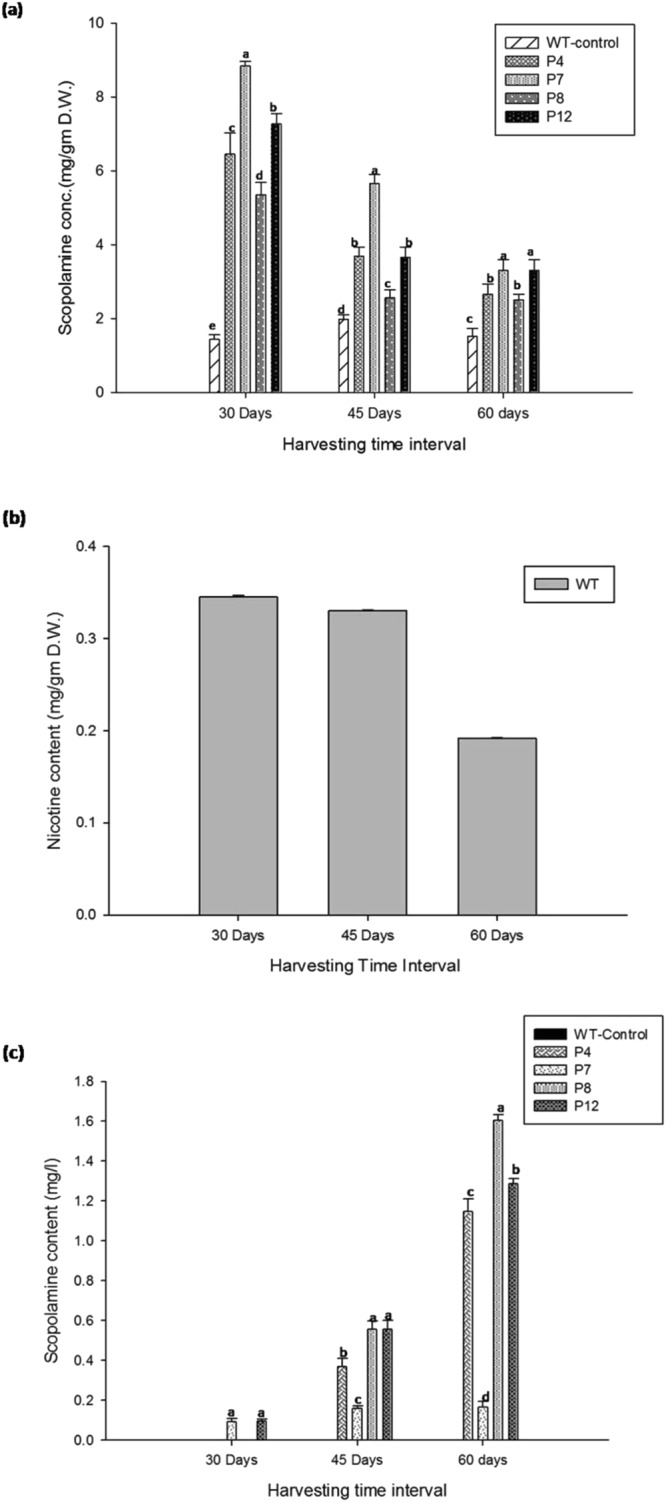
Chemical profiling of putative transgenic HR lines along with nontransgenic roots (**a**) Variation of Scopolamine content at different harvesting time interval. (**b**) Nicotine content in WT control HR. (**c**) Scopolamine content in medium at different time interval. Values are the means of three replicates ± S.D. In figure (**a**) and (**c**), bars denoted by the different letters (a–e) within same days differ significantly at p ≤ 0.05 according to Duncan’s multiple range tests.

The chemical profiling of the four hairy root lines (P4, P7, P8 and P12) through HPLC, showed that all the four were proficient in producing higher content of scopolamine than the control at 30 days after inoculation (Fig. 3a). The P7 root line revealed the maximum content of scopolamine (8.84 ± 0.117 mg/gm) which was ~6.1 fold higher than the control (1.44 ± 0.12 mg/gm). Continued cultivation up to 60 days led to a progressive decrease in the content of scopolamine (Fig. 3a), most likely due to feedback inhibition and depletion of nutrient media^20–22^. The P12 line also produced comparable amounts of scopolamine in the same time period but P8 and P4 root lines exhibited a lower content of scopolamine relative to P7 and P12 hairy root lines. However, all four hairy root lines accumulated a higher content of scopolamine than the control (Fig. 3a). These results corroborate earlier report in which accumulation of higher alkaloid was noted in the same time frame in *Duboisia*^23^. Interestingly the pyridine alkaloid nicotine was not found in any of the transgenic root lines, while the control hairy roots transformed by *A*. *rhizogenes* but lacking *QPT-RNAi* did produce nicotine (0.354 ± 0.0115 mg/gm) (Fig. 3b). This result depicts that the silencing of the *QPT* gene enhances the scopolamine biosynthesis

Leaching of scopolamine from root tissues to the medium was also analyzed. It has observed that P4 and P8 root lines, secretes maximum scopolamine in the medium at 60 days of cultivation (Fig. 3c), which indicated that the low content of scopolamine in these root lines. In contrast, leaching of scopolamine content in the culture medium in the case of P7 and P12 root lines was relatively low, as root extract of both of these clones accumulates high scopolamine content (Fig. 3c). Rationally, the release of scopolamine into the medium serves as the most significant way to downstream recovery for large-scale production which has already reported successfully^23^.

**Figure 4:**
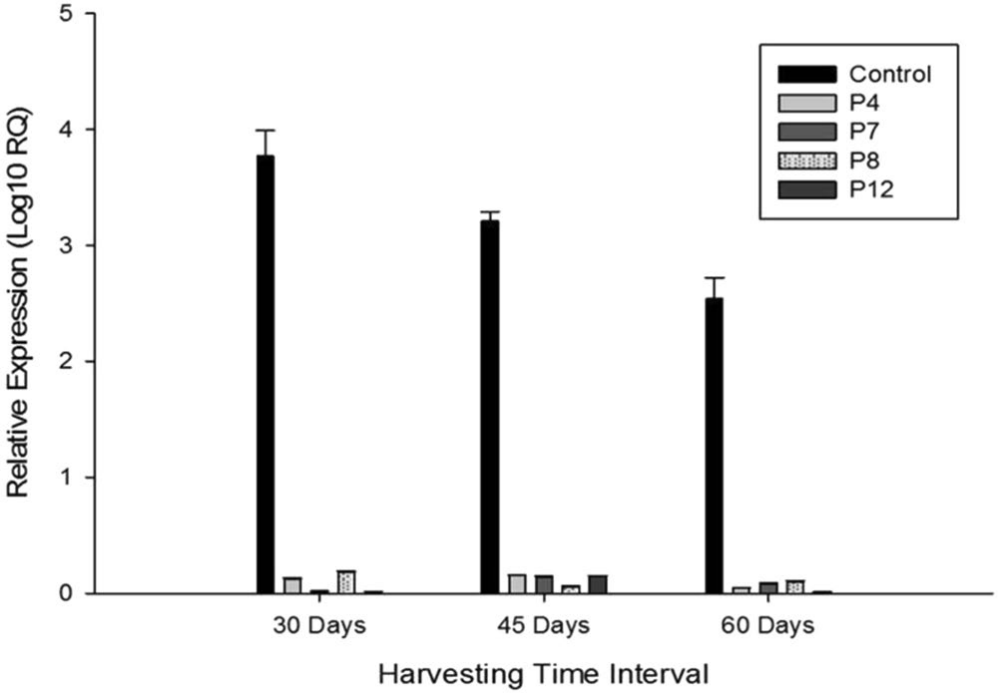
Real-time Analysis of *QPT* gene at three different harvesting time interval of HR lines.

The expression level of *QPT* gene was examined in the four hairy root lines through quantitative real-time PCR (qRT-PCR) at a different growth stage (30^th^, 45^th^ and 60^th^ days). The expression of *QPT* in all the transgenic lines had downregulated at every growth stage compared to the control hairy roots (Fig. 4). This observation was in line with the expectation that silencing of the *QPT* gene would divert methylpyrrolinium cation towards enhanced scopolamine synthesis. Although an increase in scopolamine content has reported through a repeated selection of *Duboisia myoporoides* wild-type root lines and biotransformation of *Atropa belladonna*, *D*. *leichhardtii* and *D*. *myoporoides*^2,10^. We have shown that silencing of a critical gene in the pathway could be another approach to enhanced scopolamine content in *D*. *leichhardtii* without the concomitant accumulation of nicotine and that a large amount of scopolamine would retrieve from the hairy root lines.

### Effect of methyl jasmonate elicitation on scopolamine production

**Figure 5:**
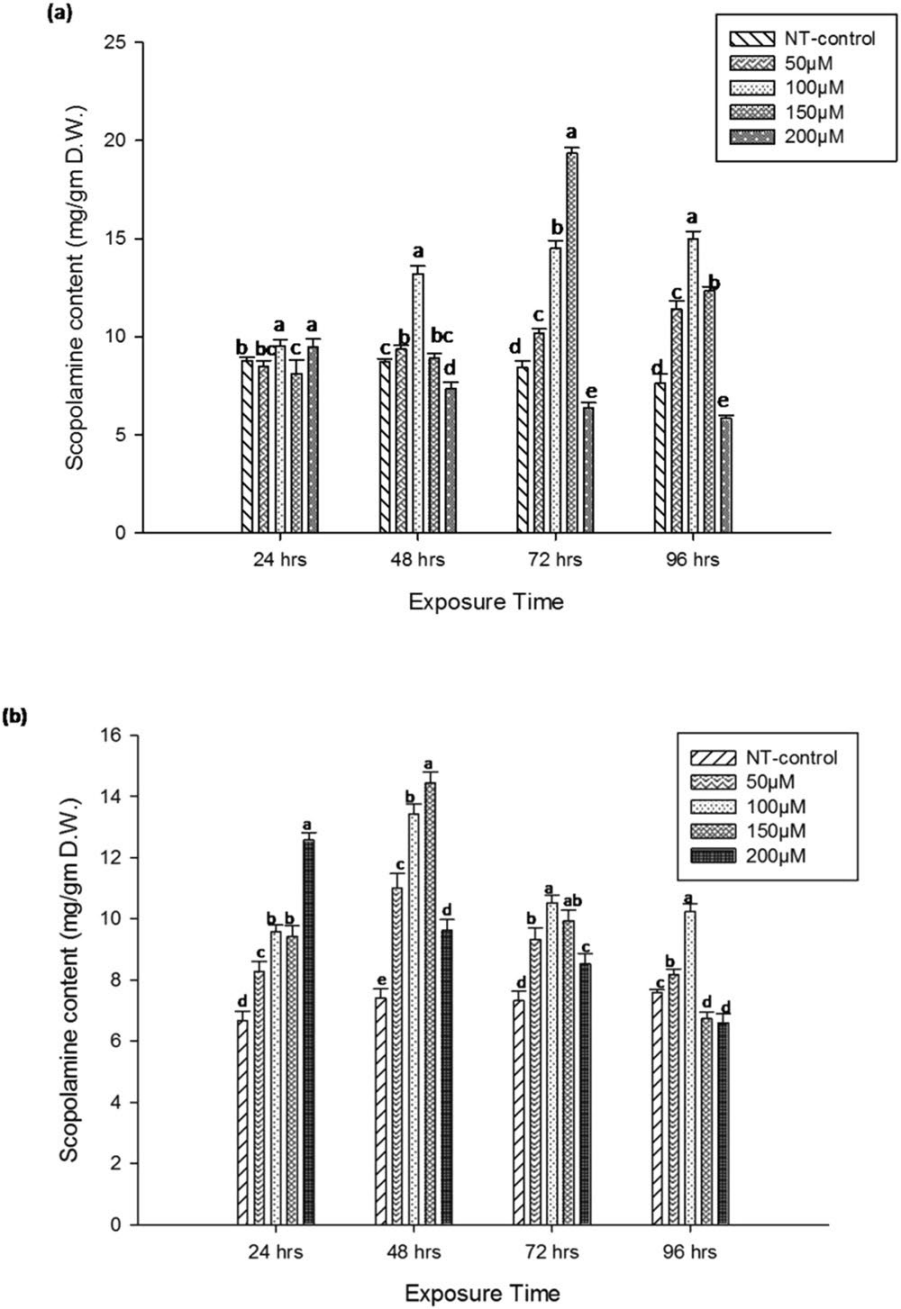
(**a**) Scopolamine content in P7 hairy root line after treatment with different concentration of MeJa. (**b**) Scopolamine content in P12 hairy root lines after treatment with different concentration of MeJa. Values are the means of three replicates ± S.D. Bars denoted by the different letters (**a**–**e**) within hours differ significantly at p ≤ 0.05 according to Duncan’s multiple range tests.

Methyl jasmonate (MeJa) is known to be involved in the signal transduction pathway to inducing particular enzymes of the biosynthetic pathways of secondary metabolites and defense compounds^24^. To examine the effect of this wound-associated hormone on scopolamine synthesis in transgenic hairy root lines (P7 and P12), micromolar concentrations of MeJa (50 µM, 100 µM, 150 µM and 200 µM) had used in culture media. In P7 root line, treatment with the lowest concentration of MeJa did not significantly increase the scopolamine content till 96 hours. With an increase in the concentration of MeJa up to 150 µM, enhanced accumulation of scopolamine content was observed (Fig. 5a). Accumulation of maximum content of scopolamine (19.344 ± 0.275 mg/gm) had found at the 96^th^ hour with 150 µM MeJa treatments which gave ∼2.2 fold higher amount than the untreated control HR and ~12.24 fold higher than the non transgenic control HR. Rationally, MeJa serves as a potent inducer of scopolamine synthesis as known from previous studies on plants such as *Scopolia parviflora*, *Datura stramonium* for improving the scopolamine and other alkaloid synthesis^25–27^^.^

Similar to P7, the P12 HR line demonstrated a two-fold higher accumulation of scopolamine (14.45 ± 0.351 mg/gm) than the untreated control and nine fold higher than the nontransgenic control with identical treatment regime but different time intervals (Fig. 5b). It is interesting to note that the accumulation of nicotine has not observed in any of the treatment which indicates that MeJa did not cause the increased level of nicotine in silenced root lines. Further increase in MeJa content and exposure time led to a decrease in scopolamine content, (Fig. 5b). This result suggested a dosage effect as known for different species^27^.

**Figure 6:**
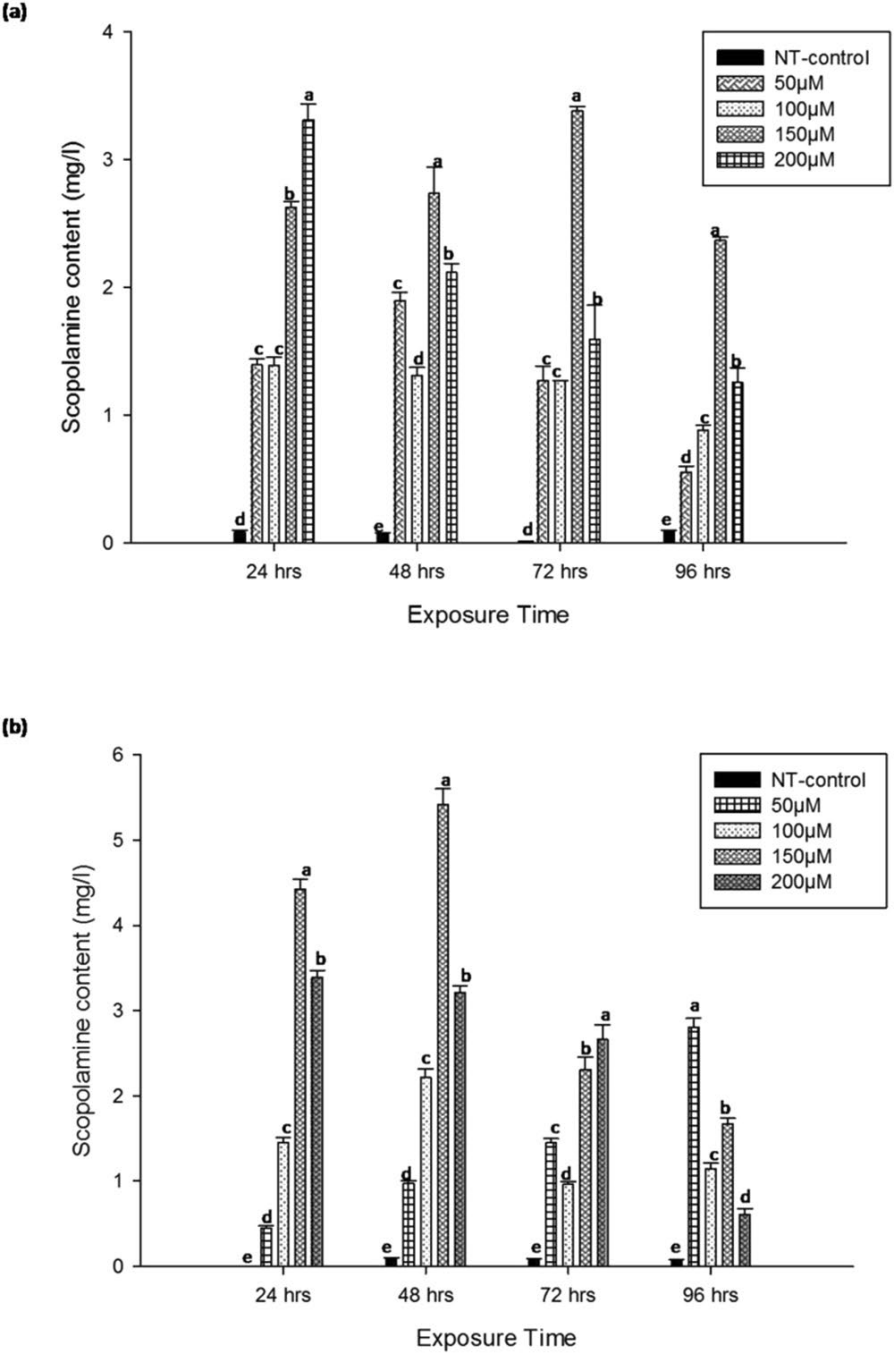
(**a**) Scopolamine content in P7 root line growing medium after treatment with different concentration of MeJa at different exposure time. (**b**) Scopolamine content in P12 root line medium after treatment with different concentration of MeJa at different exposure time. Values are the means of three replicates ± S.D. Bars denoted by the different letters (**a**–**e**) within hours differ significantly at p ≤ 0.05 according to Duncan’s multiple range tests.

It had also found that treatment with MeJa not only increased the scopolamine accumulation in hairy root tissues but also enhanced the leaching out of scopolamine in the culture medium. In P7 root line secretion of scopolamine content in medium markedly increased by treatment with MeJa relative to untreated control (Fig. 6a). Treatment with 150 µM MeJa assigns the maximum scopolamine excretion (3.38 ± 0.036 mg/l) at 72 hours of exposure, while it decreased as the exposure time increased up to 96 hours (Fig. 6a). Further treatment with 200 µM MeJa also stimulates the higher scopolamine (3.312 ± 0.123 mg/l) secretion, while an increase in exposure time resulted in decreased secretion of scopolamine in medium (Fig. 6a). Interestingly, P12 root line secreted higher scopolamine in medium with 150 µM MeJa treatment after 48 hours of exposure compared to the P7 root line (Fig. 6b). These results indicated that MeJa treatment induced the scopolamine secretion in medium, which may be due to cell membrane permeability^25^. The secretion of alkaloid in the medium due to exogenous treatment with MeJa have also reported in many plant species, like *Catharanthus roseus*, *Scopolia parviflora*^25,28^^.^

In summary, scopolamine is the most studied alkaloid in *Duboisia* species and other Solanaceous plants due to its medicinal importance and lack of synthetic occurrence^29^. *Duboisia* is the main source of scopolamine which is synthesized in the roots and translocated into shoots. Hence root is the best target for study. Since both scopolamine and nicotine have synthesized from the same intermediate methylpyrollinum cation, which reduces the scopolamine content, so it would be best to divert the methylpyrollinium cation into desired product (scopolamine) by silencing the *QPT* gene which is the master regulator of nicotine biosynthesis. However, nicotine acts as a precursor to the pyridine nucleotide cycle which is involved in the plants primary and secondary metabolism^30^. This drawback is not an obstacle in hairy root cultures. The role of the *QPT* gene was well studied previously and reported that *QPT2* gene was expressed in root tissue and involved in nicotine biosynthesis while *QPT1* gene was involved in growth and development through NAD production^31,32^. Hence the silencing of *QPT2* gene would not affect the growth of root tissues in *Duboisia leichhardtii*. We established an alternate approach to increased scopolamine synthesis and secretion based on gene silencing with the added benefit of the lack of nicotine. Treatment with an appropriate concentration of methyl jasmonate (150 µM) to specific cultivation time significantly increased the scopolamine content which could reach a commercial platform through the advent of commercial companies like Rootec® (http://www.rootec.com). It will bridge the gap in the global demand and supply of scopolamine by up-scaling of established hairy root lines which has the ultimate aim of this study

## Methods

### RNAi construct preparation

The mRNA sequences of *Nicotiana tabacum*, encoding quinolinate phosphoribosyl transferase (*NTQPT1*; GenBank: AJ748262.1 and *NTQPT2*; GenBank: AJ748263.1) had obtained from GenBank database (NCBI, USA), and primers have designed for amplification in *Duboisia leichhardtii*. Total RNA was extracted from leaves of *D*. *leichhardtii* using Spectrum plant total RNA kit (Sigma–Aldrich), and first strand cDNA was synthesized using reverse transcriptase (Takara). The expected gene was amplified using a forward primer (5′- CGTTTGGTGGATAAATGGGCGGTA-3′) and reverse primer (5′- ATCTTCAGGGAAATGTCAAGTGCTTTCAC-3′) and achieved 1047 bp of the amplicon. This PCR product was purified and cloned into a pGEMT easy vector for sequencing and aligned with *Nicotiana tabacum* sequence using multiple sequence alignment tool CLUSTAL-W. On the basis of alignment similarity, 255 bp of conserved sequence and vector pHANNIBAL were used to make an ihpRNA construct for silencing *QPT* in *D*. *leichhardtii*, similar to RNAi silencing construct of *QPT* in *N*. *tabacum*^31^. For cloning, the fragment in sense orientation in between 35 S constitutive promoter and PDK intron region *XhoI* and *Eco*RI restriction sites were added in forward and reverse primers respectively. Furthermore, for cloning the fragment in antisense orientation, *BamHI* and *HindIII* restriction sites were added in forward and reverse primers respectively and cloned sequentially as per the procedure described earlier^31^. This hairpin construct had then mobilized into the binary vector pART27 in front of the *nptII* selection marker, which subsequently mobilized into *A*. *rhizogenes* (A4) strain.

### Plant transformation and hairy root induction

*A*. *rhizogenes* strain (A4) having *QPT-*RNAi construct was grown in Yeast Mannitol Broth, supplemented with 25 mg/l kanamycin at 28 °C, 200 rpm till overnight. This bacterial culture was centrifuged for 5 min at 5000 rpm at room temperature and re-suspended in liquid MS media for the transformation of explants.

Leaves excised from *in vitro* grown *D*. *leichhardtii* plants were pricked with a needle loaded with *A*. *rhizogenes* culture, blotted dry on sterile filter paper and incubated in the dark at 25° ± 2 °C on semisolid hormone-free MS medium^33^. The explants were shifted to a hormone-free MS medium, supplemented with 250 mg/l of cefotaxime to remove the excess growth of bacteria after 48 hrs of co-cultivation. Induction of hairy roots had observed from the wounded sites of explants after two weeks of inoculation. The hairy roots were separated from the explants tissue and subcultured in the dark at 25 ± 2 °C on semisolid MS medium containing kanamycin (25 mg/l) and cefotaxime (250 mg/l). The fast-growing roots (100.7 ± 2 mg FW) were transferred to 50 ml of MS liquid medium, containing 30 g/l sucrose, in 250 ml flasks. The hairy root cultures had incubated on the rotary shaker at 75 rpm and 22 ± 5 °C temperatures under the dark condition.

### Molecular characterization of *Duboisia leichhardtii* hairy roots

The putative transformed hairy root lines had analyzed for integration of *nptII* and *rolB* gene through PCR and Southern blot analysis. Total genomic DNA of hairy root lines was isolated using CTAB method^34^. The sequence of primers used for *rolB* gene amplification was: forward primer 5′-GCTCTTGCAGTGCTAGATTT-3′ and reverse primer 5′-GAAGGTGCAAGCTACCTCTC-3′. The amplification was carried out 94 °C for 5 min as a primary denaturation, 30 subsequent cycles of 94 °C for 30 sec as a secondary denaturation, 54 °C for 30 sec as annealing, 72 °C for 1 min and 30 sec as elongation and 72 °C for 10 min as final extension. Further, amplification of the *nptII* gene was carried out as per the reported protocol previously^35^. To confirm the integration of *quinolinic acid phosphoribosyl transferase* (*QPT*) transgene into the putative transformed roots genome and its copy number, total genomic DNA was isolated from five hairy root lines. The *Pst*I digested 5.0 µg DNA from each sample along with DNA from an untransformed plant as a negative control and pART27-*QPT* as a positive control, was separated on 1% agarose gel by electrophoresis and blotted on a nylon membrane (Hybond-N+ , Amersham Pharmacia Biotech Limited, Buckinghamshire, UK). As the *QPT* gene is present within plant genome, the *nptII* gene in pART-27 had used for the preparation of probe. Random primer labeling method had used for probe preparation using 500 ng of eluted product^36^. Hybridization and washing steps were performed according to standard methods using radioactive αP^32^-dCTP nucleotide and pre-hybridization^37^. The membrane had exposed to a phosphorimager screen, and hybridization signals had detected under Typhoon 9400 Phosphorimager (GE Healthcare, Piscataway, NJ).

### Real-time quantitative PCR

To detect the expression level of *QPT* gene, total RNA had extracted from hairy root lines using Spectrum^TM^ plant total RNA kit (Sigma–Aldrich), according to the manufacturer’s instructions. For the synthesis of first strand cDNA, from 3 µg of total RNA, maxima first strand cDNA Synthesis Kit (Thermo Fisher Scientific) was used. Triplicate biological samples were used to analyze the RT-qPCR of QPT transcript using maxima SYBER green/ROX qPCR (Thermo Fisher Scientific). The reaction volume of RT-qPCR was ten µl which include five µl maxima SYBER green, forward and reverse primers (300 nM each) and diluted cDNA as a template. PCR conditions had kept according to the previously reported protocol^38^. The threshold (Ct) value for the *QPT* gene had normalized against the Ct value of *Duboisia* species housekeeping gene actin. Mean Ct values had calculated from technical triplicates, and the relative levels of the transcript of *QPT* of transgenic hairy roots were compared with wild-type hairy roots (calibrator) using the relative quantification 2−ΔΔCt method^39^. Fluorescent signal intensities were recorded and analyzed on an Applied Biosystems StepOnePlusTMReal-Time PCR System.

### Study of growth kinetics and phytochemical extraction of hairy root lines

Four randomly selected root lines (viz. P4, P7, P8, and P12) were subcultured (100.7 ± 2 mg 50 ml^−1^) on liquid MS media supplemented with 25 mg /l of kanamycin for growth kinetics study. All the root lines and media had harvested at a different time intervals (i.e., 30^th^, 45^th^ and 60^th^ days) and roots were air dried at room temperature (25 °C –  30 °C) for three days. The final and initial weight of hairy roots had recorded for growth kinetics study and growth index was measured according to the formula [GI = (final weight − initial weight) initial weight) ×100. The dried hairy roots tissues were finely ground and extracted according to reported protocol^10^. To find the leaching of scopolamine and nicotine in the growth media, the culture media of all the root lines had also extracted in a respective time interval.

### HPLC analysis of scopolamine and nicotine alkaloids

The crude root extracts were dissolved in 1 ml of HPLC grade methanol, followed by filtration through a 0.22 µm syringe filter (Millipore Corporation, Billerica, MA). The filtered samples had injected to Waters (Milford MA, USA) HPLC system equipped with a binary pump, manual injector, photodiode array detector (PDA, model 996), Empower Pro software (Waters, USA) was used for the quantification. Reverse Phase Column of Sunfire C18 (4.6 mm × 250 mm, 5 µm coating; Waters, USA) had used for the separation and quantification of scopolamine and nicotine. The gradient elution was carried out using a solvent system comprising acetonitrile (solvent A) and phosphate buffer (solvent B, pH 6.5) with a constant flow rate of 1.0 ml/min throughout the 40 min analysis. A gradient elution programming had performed with 10% A and 90% B for initial 15 min then 10% A to 75% A for 15–24 min. This flow continued up to 35 min, and then the initial flow was attained as 10% A and 90% B for the next 5 min. For quantitative analysis, the system had calibrated with standards of nicotine and scopolamine obtained commercially (Sigma–Aldrich, USA) and the peak was detected at 260 nm for nicotine and 205 nm for scopolamine. For quantification of Nicotine and Scopolamine content, the retention time and data obtained from the standards were compared with that of samples and then calculated on the basis of the peak area of the analyte in the samples and the peak area of the standards.

### Elicitation with methyl jasmonate

The 28^th^ days old transgenic hairy root lines (P7 and P12) of *D*. *leichhardtii* grown in liquid MS media, were treated with various concentrations of MeJa (50 µM, 100 µM, 150 µM and 200 µM). All culture experiments had performed in triplicate. After treatment with MeJa (Sigma Aldrich), all the culture flask had incubated on a rotary shaker at 75 rpm and 22 ± 5 °C temperature under the dark condition. Roots from all treatments, as well as the culture medium, were harvested after 24 hrs, 48 hrs, 72 hrs and 96 hrs of the treatment of both hairy roots lines (P7 and P12). Further, all the dry roots were extracted for analysis of scopolamine and nicotine production as mentioned above.

### Statistical analysis

All culture experiments were carried out in triplicate and data were analyzed by analysis of variance (ANOVA)., statistical significance had analyzed with the software SPSS 16.0 (SPSS Inc.USA) to detect the significant differences between means. The probability level was compared at a p < 0.05 using Duncan’s multiple range tests.

## Electronic supplementary material

Supplementary Information
