# Supplementary material for: Silencing of quinolinic acid phosphoribosyl transferase (QPT) gene for enhanced production of scopolamine in hairy root culture of Duboisia leichhardtii
Source: Sci Rep. 2018 Sep 17;8:13939. doi: 10.1038/s41598-018-32396-0 (PMC6141460; doi:10.1038/s41598-018-32396-0)
Supplement: Supplementary file 1 — Supplementary Information [file 41598_2018_32396_MOESM1_ESM.pdf]

Silencing of quinolinic acid phosphoribosyl transferase (*QPT*) gene for enhanced production of scopolamine in hairy root culture of *Duboisia leichhardtii*

Pooja Singh[1],[2], Ratnum Prasad[1], Rashi Tewari[3], Meraj Jaidi[4], Susheel Kumar[4], P.K. Rout[3] and Laiq ur Rahman1*

[1]Plant Biotechnology Division, Central Institute of Medicinal and Aromatic Plants (CSIR-CIMAP), P.O. CIMAP, Picnic Spot Road, Lucknow, U.P. 226015, India

[2]Academy of Scientific and Innovative Research (AcSIR), CSIR-Central Institute of Medicinal and Aromatic Plants, Lucknow 226015, India

[3]Chemical Science Division, Central Institute of Medicinal and Aromatic Plants, P.O. CIMAP, Picnic Spot Road, Lucknow, U.P. 226015, India

[4]Plant Molecular Virology Laboratory, CPMB Division, National Botanical Research Institute, Lucknow, U.P. 226001, India

*Corresponding author: e-mail: l.rahman@cimap.res.in

Tel.: +91 522 2718555; fax: +91 522 2342666.

Fig. S1

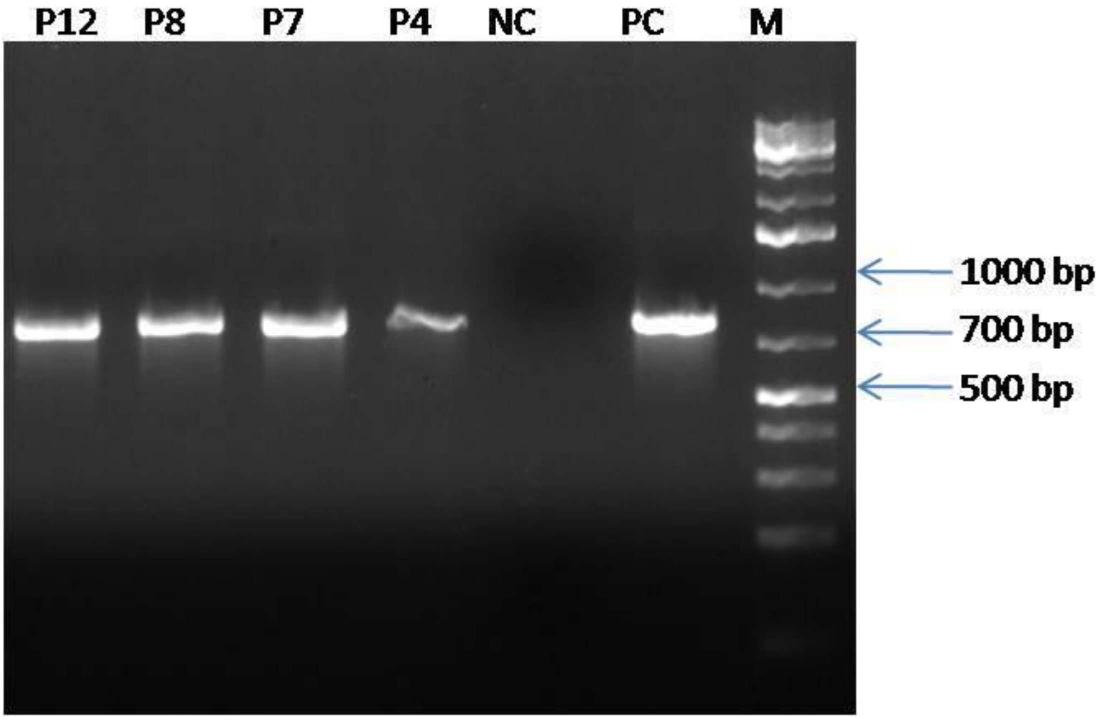

Fig.S2

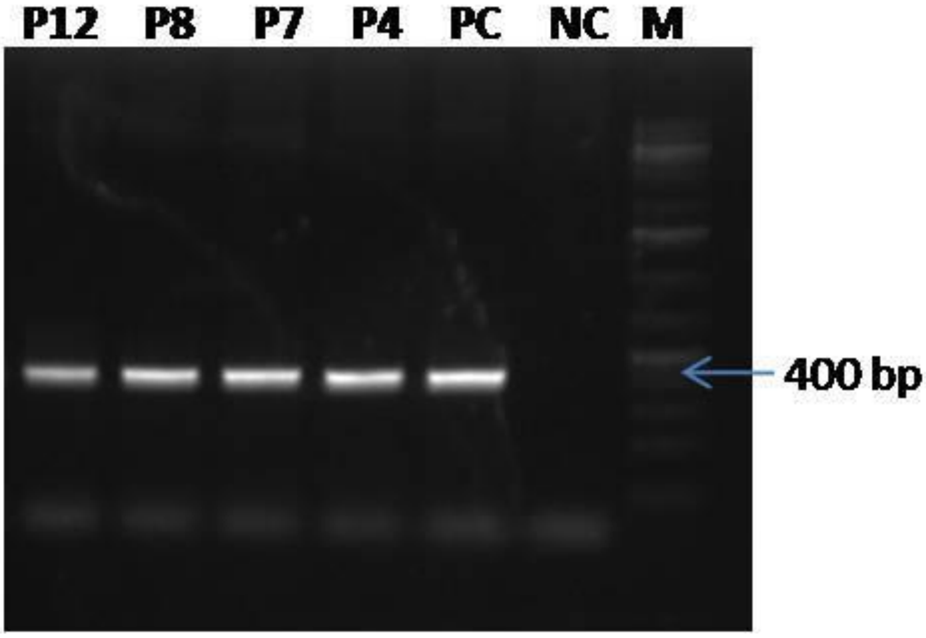

Fig. S3

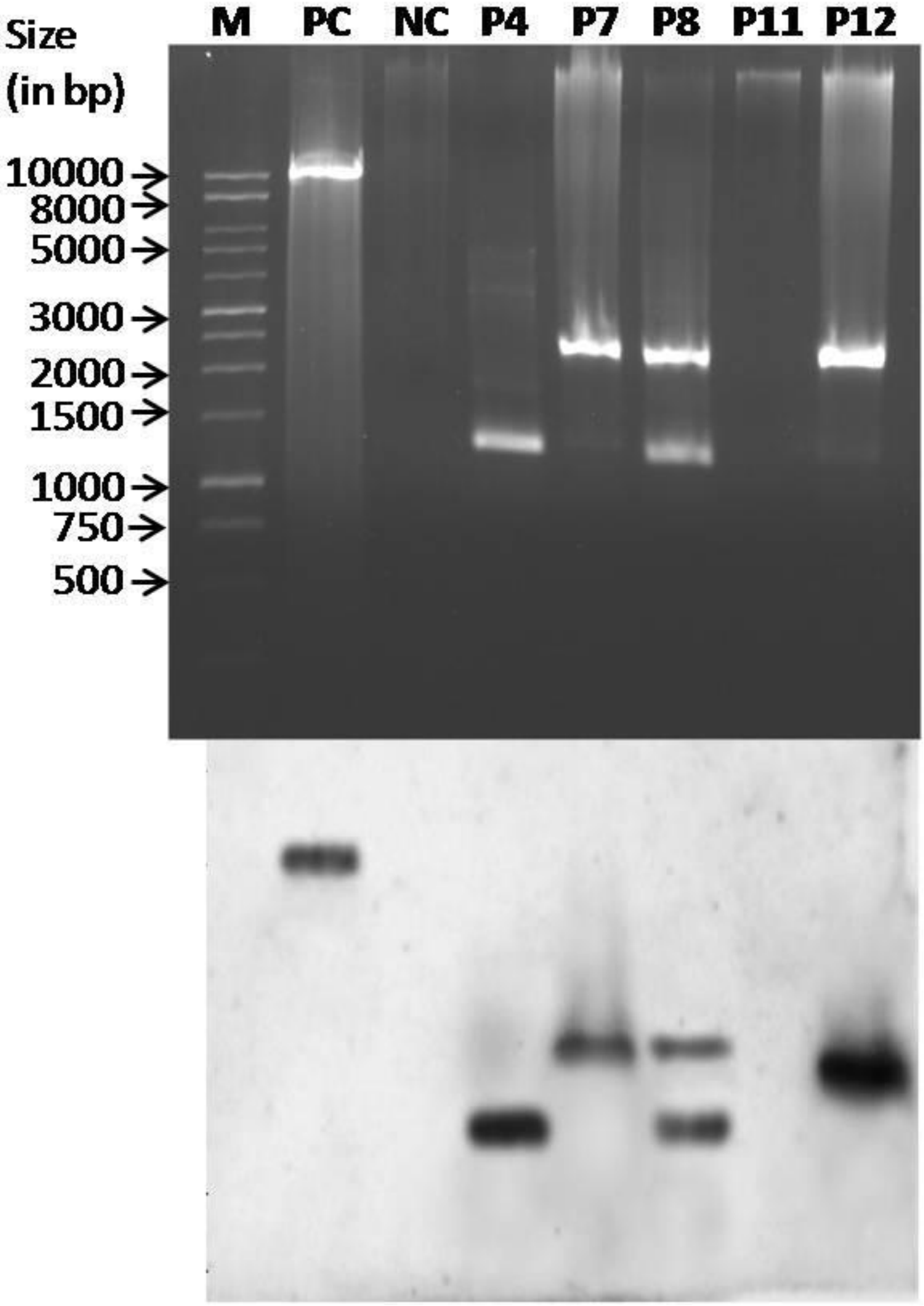
M PC NC P4 P7 P8 P11 P12
Size
(in bp)
10000
8000
5000
3000
2000
1500
1000
750
500

Fig. S4

```
clustalw.aln

CLUSTAL 2.1 multiple sequence alignment


Nicotiana-tabacum                  MFRAIPFTATVHPYAITAPRLVVKMSAIATKNTRVESLEVKPPAHPTYDL
pGEMT_easy_vector_cloned_seque     MFKVLPFTAIVHPYAITAPRLVKMSAIATKNATVESLAVKAPPAHPTYNL
                                   **:.:**** ************    :  :.: *   .    *******:*

Nicotiana-tabacum                  KGVMQLALSEDAGNLGDVTCKATIPLDMESDAHFLAKEDGIIAGIALAEM
pGEMT_easy_vector_cloned_seque     KGVIQLALSEDAGNLGDVTCKATIPVDMESEAHFLAKEDGIVAGIALAEM
                                   ***:*********************:****:**********:********

Nicotiana-tabacum                  IFAEVDPSLKVEWYVNDGDKVHKGLKFGKVQGNAYNIVIAERVVLNFMQR
pGEMT_easy_vector_cloned_seque     IFAEVDPSLKVEWSIKDGDIVHKGLKFGKVQGNAHSIVIAERVVLNFMQR
                                   *************  ::*** ***************:.***************

Nicotiana-tabacum                  MSGIATLTKEMADAAHPAYILETRKTAPGLRLVDKWAVLIGGGKNHRMGL
pGEMT_easy_vector_cloned_seque     MSGIATLTKAMADAAHPAYILETRKTAPGLRLVDKWAVLIGGGKNHRMGL
                                   ********* ****************************************

Nicotiana-tabacum                  FDMVMIKDNHISAAGGVGKALKSVDQYLEQNKLQIGVEVETRTIAEVREV
pGEMT_easy_vector_cloned_seque     FDMVMIKDNHISAVGGVGKALKSVDQYLEQNKLQMIQQVETRTIAEVREV
                                   *************.********************:  :*************

Nicotiana-tabacum                  LDYASQTKTSLTRIMLDNMVVPLSNGDIDVSMLKEAVELINGRFDTEASG
pGEMT_easy_vector_cloned_seque     LDYASQTKTSLTRIMLDNMVVPLSNGDIDVSMLKEAVELINGRFDTEVTP
                                   ***********************************************.:

Nicotiana-tabacum                  NVTLETVHKIGQTGVTYISSGALTHSVKALDISLKIDTELALEVGRRTKR
pGEMT_easy_vector_cloned_seque     GFIFINYFTRVNYVARTLQICSRCPNAFCEST------------------
                                   .. : . ..  :  .  :.  :   .. . .

Nicotiana-tabacum                  A
pGEMT_easy_vector_cloned_seque     -
```

Fig.S5

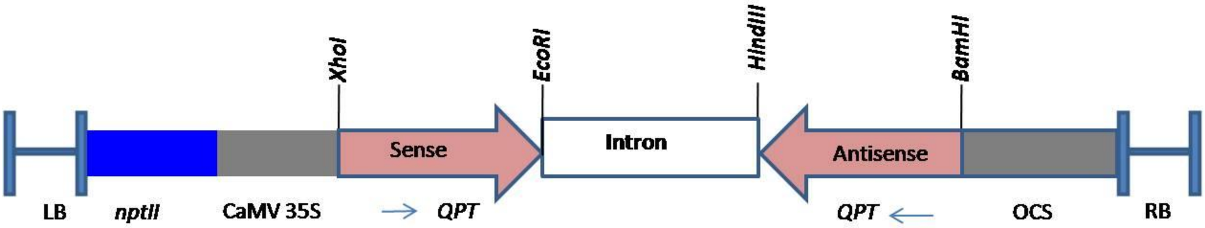

AGGCAATGGCAGATGCTGCACACCCTGCTTATATCTTGGAGACAAGGAAAACTGCTCCAGGATTACGTTTGGTGGATAAATGGGCGG
TATTGATTGGCGGGGGAAAGAATCACAGGATGGGCTTATTTGATATGGTAATGATAAAAGACAATCACATATCTGCTGTTGGAGGTGT
CGGCAAAGCTCTAAAATCTGTGGATCAGTATTTGGAGCAAAATAAACTTCAAATGATCCAACAGGTTGAAACCAGGACAA

Legends

Fig. S1. PCR amplification of the *nptII* gene produced 786 bp of the amplicon. M -marker (1 kb plus ladder), PC-Positive Control, NC-Negative Control. P4, P7, P8 & P12 are the putative transformants. (uncropped image).

Fig. S2. PCR amplification of *rol* B gene produced 400bp of the amplicon. M marker 1 kb plus ladder, PC-positive control, NC -Negative Control, P4, P7, P8 & P12 are the putative transformants. (uncropped image).

Fig. S3. Gel image with respective southern blot of putative transgenic hairy root lines. M-marker (1 Kb ladder), PC-Positive Control, NC- Negative Control, P4, P7, P8, P11, and P12 are putative transgenic hairy root lines.

Fig. S4. Multiple sequence alignment of *Nicotiana tabacum* and cloned sequence in a pGEMT easy vector. The amino acid sequence which represented with asterisk showed the most identical, sequence show with double dot represents the most conserved region, while a single dot represents the identical region.

Fig. S5. Schematic representation of the QPT hairpin (hpRNAi) construct map. Sequence highlighted with pink color, cloned in sense and antisense orientation with the respective restriction enzyme which shown in construct map at 5’ end of forward and reverse primers.
